# Our New York Letter

**Published:** 1886-02

**Authors:** Wm. Penny

**Affiliations:** New York


### OUR NEW YORK LETTER.

NEW YORK, December 10, 1885.

Editor Daniel's Medical Journal:

*Sir:*—I have another request for a postponement of my letter on Gynecology. There is so much here of interest in surgery that I like to jot it down while it is fresh, besides the subject of gynecology is a good deal like the large flowered goods that was worn by gentlemen a good many years since. A young swell, who had admired a piece of this kind that was displayed in a tailor's window, requested his measure to be taken for a pair of pants, to be made from the same, and to whom the tailor replies, with the dignity of his profession; "Sir, it takes five pair to cut into the pattern, and we can't take an order for less." So it is with gynecology; there are so many in it, and such a demand for tickets for operations, that the process of working it up is slow. Besides, there is very little that is new in gynecological surgery, while in general surgery there is a great deal that is fresh. New operations are being devised and old ones revived and modified, and, if possible, the name of a German surgeon tacked on to it then.

Among the old surgical operations that have been revived is supra pubic operation for stone in the bladder, and lest some of your readers may be misled by my saying that it is an old operation revived, let me here make the distinction; the old operation was intra peritoneal, the new is extra peritoneal. I was present at a supra pubic operation for stone performed by Dr. Pilcher. The steps of the operation are as follows: After the patient was anæthetized and the parts shaved, washed and disinfected, a Nelaton's catheter was introduced into the bladder and the urine drawn off; a tape was tied around the penis to prevent fluid from escaping around the catheter; a soft rubber bag was introduced into the rectum, and twelve ounces of water pumped into this receptacle; then after, securing the tube connected with it, the stop-cock was removed and connected with the catheter, when six or eight ounces of water was pumped into the bladder, which was now distinctly visible, or at least the bulging of the abdominal wall.

The incision was made in the linea alba about three inches in length; after all bleeding points had been ligated, a narrow strip was dissected from the anterior wall of the bladder down to the mucous membrane, and about one and a half inches in length. A silk suture was passed through the muscular coat at each end of the incision so as to be able to control the bladder.

The incision into the bladder was then made with a common scalpel, and as the bladder was firmly held against the abdominal wound by both, the tension on the sutures and the presure from the bag in the rectum, leakage into the pelvic space was practically impossible.

The stone was readily reached and removed with a pair of ordinary polypus forceps. The bladder was then explored with the finger, and the wound was closed with gut ligtures, care being taken that they did not penetrate the mucous membrane. After removing the silk sutures and allowing the water to escape from the rectal bag, the external wound was closed with wire sutures, and dressed in the usual antiseptic manner.

It will be seen that a very small addition to the surgeon's ordinary case will be required. This addition will consist of the rectal bag, a small stop-cock, and a Nelaton's catheter; he being supposed to have a pair of scissors that are curved on the flat.

As a contrast to this, I saw, a few days later, Dr. Bull perform Bigelow's operation of lithoplaxy. Nothing could be stronger than the contrast between these two operations; the extreme simplicity of the instruments required in the one, and the imposing armamentarium required in the other. In the one operation every step was in sight, and no extraordinary surgical skill required—in the other a remarkable amount of dexterity is required. Both of these operators are remarkably dextrous; but from the extreme dissimilarity of the operations, no comparison could be made, even if I felt so disposed.

In comparing the supra pubic operation with lithotomy and lithoplaxy (lithotroty being no longer in vogue), it has certain very great advantages over both of these. Neoplasms and incrustations can be removed by this operation that could not be, by any other. The interior of the bladder can be inspected, and bleeding points ligated with the utmost facility. The absence of danger from the passage of urine over a raw surface, as occurs in lithotomy, and probably, also, in lithoplaxy, is greatly in its favor, and the certainty of getting all the stone is another.

In the case of Dr. Bull's but a small amount of the debris could be evacuated, even after repeated attempts, as the stone was soft, and thoroughly pulverized; it probably made no particular difference, but it might.

The fashionable operation here is for cancer of the tongue. It is a little singular, since the death of General Grant, how frequent these cases have become. I have witnessed the operation three times in three weeks; how often it has been performed here, I have no idea.

The steps in the operation are, after etherization: First, tracheotomy; second, tamponing the pharynx; third, the external incision just below the margin of the submaxilary gland, and extending from the ramus of the jaw to the middle of the chin. The incissions are made layer by layer, and the veins and arteries are ligated as found; fourth, extirpation of the submaxilary gland, and cutting through the mucous membrane; this gives a large opening; fifth, the tongue is pulled out, split down the middle, and cut off near the root, then returned to the mouth; sixth, a stomach tube is introduced and the stomach is washed out; seventh, the external wound is closed *a la carte*; eighth, the mouth is stuffed with iodoform gauze and a bandage applied.

How any person can stand it to have their pharynx plugged, and their mouth stuffed with iodoform gauze for eight or ten days, I can't conceive; "but great is Diana of Ephesus." The operation is a showy one, and brings down the house.

How many ulcers of the tongue, due either to defective teeth or the sequellæ of syphilis I have treated, it might look like boasting to say, and if any of them resulted in cancer I am not aware of it. Of course there is cancer of the tongue, and General Grant died of it, but it has been until recently a rare disease.

Wm. Penny, M. D.

P. S.—Will give you the letter on gynecology next.

